# Plasma neuropeptide Y: a biomarker for symptom severity in chronic fatigue syndrome

**DOI:** 10.1186/1744-9081-6-76

**Published:** 2010-12-29

**Authors:** Mary A Fletcher, Martin Rosenthal, Michael Antoni, Gail Ironson, Xiao R Zeng, Zachary Barnes, Jeanna M Harvey, Barry Hurwitz, Silvina Levis, Gordon Broderick, Nancy G Klimas

**Affiliations:** 1 Department of Medicine, University of Miami Miller School of Medicine, 1600 NW 10th Ave, Miami, FL USA; 2 Department of Psychology, University of Miami, 5665 Ponce DeLeon Blvd, Coral Gables, FL USA; 3 Miami Veterans Administration Health Care Center, 1201 NW 16th St, Miami, FL USA; 4 Department of Medicine, University of Alberta, Canada

## Abstract

**Background:**

Chronic fatigue syndrome (CFS) is a complex, multi-symptom illness with a multisystem pathogenesis involving alterations in the nervous, endocrine and immune systems.

Abnormalities in stress responses have been identified as potential triggers or mediators of CFS symptoms. This study focused on the stress mediator neuropeptide Y (NPY). We hypothesized that NPY would be a useful biomarker for CFS.

**Methods:**

The CFS patients (n = 93) were from the Chronic Fatigue and Related Disorders Clinic at the University of Miami and met the 1994 case definition of Fukuda and colleagues. Healthy sedentary controls (n = 100)) were from NIH or VA funded studies. Another fatiguing, multi-symptom illness, Gulf War Illness (GWI), was also compared to CFS. We measured NPY in plasma using a radioimmunoassay (RIA). Psychometric measures, available for a subset of CFS patients included: Perceived Stress Scale, Profile of Mood States, ATQ Positive & Negative Self-Talk Scores, the COPE, the Beck Depression Inventory, Fatigue Symptom Inventory, Cognitive Capacity Screening Examination, Medical Outcomes Survey Short Form-36, and the Quality of Life Scale.

**Results:**

Plasma NPY was elevated in CFS subjects, compared to controls (p = .000) and to GWI cases (p = .000). Receiver operating characteristics (ROC) curve analyses indicated that the predictive ability of plasma NPY to distinguish CFS patients from healthy controls and from GWI was significantly better than chance alone. In 42 patients with CFS, plasma NPY had significant correlations (<0.05) with perceived stress, depression, anger/hostility, confusion, negative thoughts, positive thoughts, general health, and cognitive status. In each case the correlation (+ or -) was in the anticipated direction.

**Conclusions:**

This study is the first in the CFS literature to report that plasma NPY is elevated compared to healthy controls and to a fatigued comparison group, GWI patients. The significant correlations of NPY with stress, negative mood, general health, depression and cognitive function strongly suggest that this peptide be considered as a biomarker to distinguish subsets of CFS.

## Background

According to the 1994 international research case definition [1], as modified according to Reeves, et al [2], chronic fatigue syndrome (CFS) is an illness characterized by 1) the presence of persisting, debilitating fatigue that does not resolve with bed rest, and lasts for at least six months resulting in severe impairment in daily function; 2) by symptoms and disability that cannot be ascribed to any other medical and psychiatric conditions. Diagnosis relies in large part on behavioral markers, either patients' self-reported symptoms or observations by clinicians. Laboratory diagnostic and screening tests are not widely available.

In 60 to 80% of published samples, CFS presented with acute onset of illness, with systemic symptoms similar to influenza infection that did not subside [3]. The sudden onset, the symptoms of myalgia, arthralgia, sore throat and tender lymphadenopathy support theories of infection induced illness [3,4]. Published reports both confirm and deny associated microbial infections, reactivation of latent herpes virus infections and/or retrovirus infections in CFS [5-14].

CFS is responsible for significant morbidity and occurs in an estimated 0.42% of people, predominately female, in the United States and worldwide [15,16]. Prior work strongly suggests this complex, multi-symptom illness has a multi-system pathogenesis that involves the nervous, endocrine and immune systems [3,17-21]. Abnormalities in stress responses have been identified as potential triggers or mediators of CFS symptoms [22-24].

Neuropeptide Y (NPY) is found in both the central and peripheral nervous systems. In the peripheral nervous system, NPY is concentrated in and released from sympathetic nerve endings, either alone or with catecholamines. Irwin reported NPY increased in depressed and stressed subjects, and suggested that "circulating levels of this neuropeptide serves as a tonic measure of sympathetic activity"[25]. NPY release follows stress such as strenuous exercise [26], panic disorders [27], and cold exposure [28]. In the periphery, NPY is activating and stimulates the stress response, but in the brain, NPY is anxiolytic, inhibitory of sympathetic activity and causes lowering of blood pressure and heart rate [29,30]. Concentrations of NPY in the brain are higher than other neuropeptides, particularly in the limbic system, including the amygdala and the hypothalamus, all areas of the brain involved with emotion [31,32].

Plasma levels of NPY are reported to be elevated in other complex multi-symptom illnesses associated with immunologic dysfunction, including rheumatoid arthritis (RA) and systemic lupus erythematosus (SLE) [33]. A recent study compared 51 fibromyalgia patients to 25 healthy controls and reported elevated plasma NPY [34]. However, an earlier study found lower NPY in 12 fibromyalgia cases [35]

Given these reports, it seemed likely that plasma NPY would be elevated in CFS. Elevated NPY would be anticipated to correlate with psychological measures of stress and other aspects of CFS symptoms. However, we could find no such studies. There is a clear need for CFS biomarkers that are useful in diagnosis, in defining patient subgroups and in therapeutic trials. We tested and confirmed the hypotheses that elevation of peripheral NPY occurs in CFS and that elevation of NPY is associated with severity of stress, negative mood and clinical symptoms.

## Methods

### Objectives

Abnormalities in stress responses have been identified as potential triggers or mediators of CFS symptoms. This study focused on the stress mediator neuropeptide Y (NPY). The aim was to examine the potential of plasma NPY as a biomarker for stress and symptom severity in CFS.

### Participants

All subjects were participants in research studies taking place between 2002 to 2010 at the University of Miami or the Miami Veterans Administration Health Care Center (funded by NIH, VA, Chronic Fatigue and Immunodeficiency Syndrome Association or the University of Miami).

Chronic fatigue syndrome patients (n = 93; age 18 to 60, mean age 44 +/- 9 S.D.; median age 46, 25 percentile 38, 75 percentile 50; 83% female) were drawn from the University of Miami Miller School of Medicine CFS and Immunodeficiency Clinic after they were diagnosed with CFS, using the 1994 international research case definition [1], as modified according to Reeves, et al [2]. This requires 4 of 8 symptoms including exercise induced relapse, myalgia, arthralgia, headache of a new and different type, nonrestorative sleep, cognitive complaints, sore throat and tender lymph nodes. Exclusion criteria included any active medical condition that could explain the presence of chronic fatigue, including diabetes, the current use of immunomodulatory or antibiotic medications, and a past or present psychiatric diagnosis of psychosis (e.g., schizophrenia), dementia, major depressive disorder with psychotic or melancholic features, bipolar disorder, anorexia or bulimia nervosa, or alcohol/substance abuse disorder within two years of the onset of the fatigue or anytime thereafter. In agreement with published samples, the majority of CFS cases presented with a history of acute onset of illness, with systemic symptoms similar to influenza infection that did not subside [4].

Sedentary healthy controls, with no active medical or psychiatric conditions, immunomodulating medications or alcohol/substance abuse, (n = 100; 84% female; mean age 41 +/- 10 S.D.; median age 41, 25 percentile 35, 75 percentile 49) were from NIH or VA funded studies conducted by one or more of the investigators in this study.

A comparison group of patients with a fatiguing, multi-symptom illness, GWI, was also included in the study (N = 37; 29% female, mean age 44 +/- 7; median age 42, 25 percentile 38, 75 percentile 48). The inclusion criteria for GWI, derived from the case definition by Fukuda et al [36], was: a veteran deployed to the theater of operations for Operation Desert Storm between August 8, 1990 and July 31, 1991 with one or more symptoms present greater than 6 months from at least 2 of the following: fatigue; mood and cognitive complaints; and musculoskeletal complaints (joint pain, stiffness or muscle pain). These subjects had no active medical or psychiatric conditions, immunomodulating medications or alcohol/substance abuse.

### Blood collection

Morning, non-fasting blood samples were collected, prior to the collection of clinical or and psychometric data. The blood was collected into ethylenediaminetetraacetic acid and all of the samples for this study were delivered to University of Miami clinical immunology laboratory within 2 hours. The laboratory received and assayed the samples in a blinded fashion. Plasma was separated from cells within 2 hours of collection, aliquoted into cryovials and stored at - 80°C until assayed. The length of time of storage at -80°C varied from 6 months to 5 years for the samples in this study. Samples were thawed only one time.

### Assay of NPY

Plasma NPY was measured by direct assay without extraction using a competitive radioimmunoassay (RIA) from ALPCO Diagnostics (Salem, NH). The antiserum was raised against synthetic NPY conjugated to bovine thyroglobulin and cross-reacted less than 2.0% with human peptide YY. The NPY in standards and samples competed with ^125^I-labeled NPY in binding to the antibody. ^125^I-NPY bound in reverse proportion to the concentration of NPY in standards and samples. Antibody-bound ^125^I-NPY was separated from the unbound fraction with a double antibody coupled to the solid phase. The radioactivity of the antibody-bound ^125^I-NPY was then determined. The measurable range of the assay was 9.4 - 300 pmol/L and sensitivity was 3 pmol/L. The manufacturer reported a mean value of 74 pmol/L (SD = ± 15) and a range of 36-120 pmol/L for 109 healthy controls (ages 20-60 years). The intra assay coefficient of variation (CV%) was 4.75%. The inter assay CV% was 8.4%. All samples were assayed in duplicate. All assays were done in the same laboratory. Individual runs were mixtures of samples from the 3 cohorts in the study. A set of control samples were included in each assay.

### Self report measures of clinical symptoms

On a subset of 42 CFS cases in this study, perceptions of general health and well-being, severity of clinical symptoms of fatigue, stress, cognitive difficulties, psychological distress and maladaptive coping were assessed using the following self-report measures:

• The Medical Outcomes Survey Short Form-36 (SF-36) assessed health-related quality of life including: limitations in social activities, emotional problems and general health [37].

• The Perceived Stress Scale (PSS) was used as a measure of stress [38].

• The Fatigue Severity Inventory (FSI) was designed for measuring fatigue severity. Items in the inventory are statements related to fatigue perceptions [39].

• The COPE, a multidimensional coping inventory, was used to assess the different ways in which people respond to stress [40]. Subscales for denial, behavioral disengagement and self-blame were used.

• The Quality of Life Scale (QOL) total score measured general well-being, cognitive functioning, affective status, physical health status and social activity [41].

• The Profile of Mood States (POMS) a measure of psychological distress consists of 65 adjectives rated on a 0-4 scale that are comprised of subscales measuring 'depression-dejection', 'tension-anxiety', 'anger-hostility', 'confusion-bewilderment', 'vigor-activity' and 'fatigue-inertia' [42].

• The Adult Temperament Questionnaire (ATQ) is a 77-item self-report instrument used for assessment of positive and negative thoughts and is a measure of str*e*ss that is possibly predictive of stress-related health problems [43].

• The Cognitive Capacity Screening Examination (CCSE) was used to quantify the general cognitive status of subjects [44].

• The Beck Depression Inventory (BDI) is a 21-question multiple-choice self-report inventory used to measure severity of depression [45].

These instruments were completed by the CFS patients on the same visit as the blood draw.

### Ethics statement

This study was approved by the University of Miami Institutional Review Board. All subjects signed a written informed consent approved by the University of Miami Institutional Review Board. All consented to blood draw and to the availability of the stored samples for additional bioassays. Participants were English speaking with at least an 8th grade education to ensure they were able to comprehend the informed consent as well as read and complete the questionnaires.

### Statistical methods

The Mann-Whitney U test was used to determine the magnitudes of between-group (case-control) differences. Pearson correlations were calculated for the psychosocial measures of disease severity and plasma NPY in 42 CFS cases. Values of p < 0.05 were considered statistically significant. The diagnostic accuracy of NPY was assessed in terms of true positive (sensitivity) versus true negative (1-specificity) using nonparametric receiver operating characteristics (ROC) analyses [46] available in the Statistical Package for Social Sciences (SPSS) software for Windows (SPSS Inc, Chicago, IL). The nonparametric ROC plot uses all of the data, makes no parametric assumptions and provides unbiased estimates of sensitivity and specificity, indicating the ability of a test to discriminate between two alternate states of health, in this case, CFS cases and healthy controls and CFS cases and GWI cases. The calculation of the area under the curve (AUC) provided a convenient way to determine the ability of the NPY assay to distinguish these groups. An AUC = 0.5 indicates that the test shows no difference between the two groups while AUC = 1.0 is found if the test gives a perfect separation between groups.

## Results

**Figure 1:**
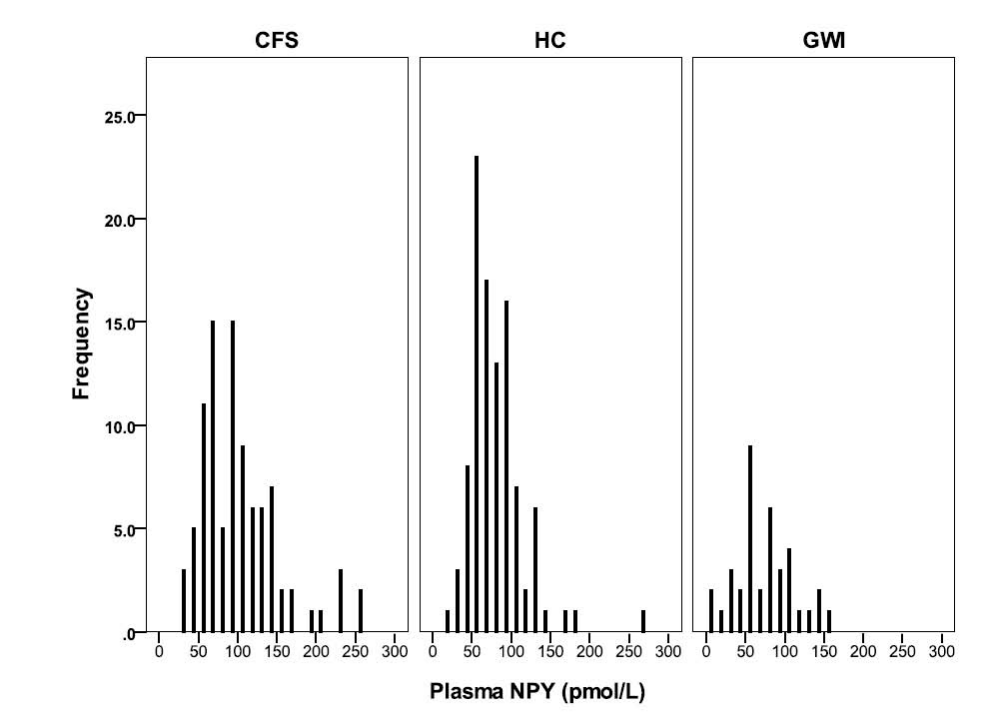
**The panels show the histogram of plasma NPY values for the Chronic Fatigue Syndrome cases (CFS), the healthy controls (HC) and the Gulf War Illness cases (GWI)**.

**Figure 2:**
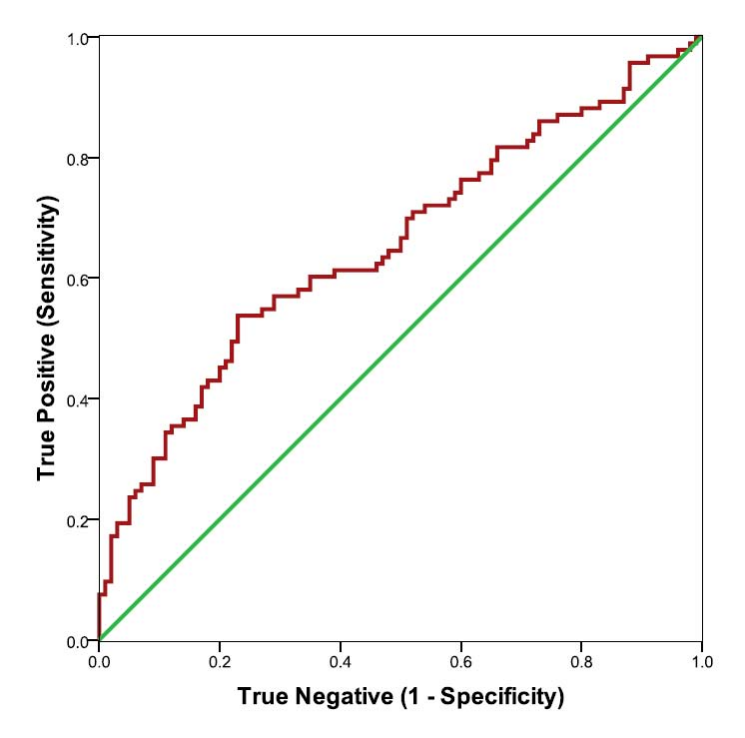
**ROC curve analyses of plasma NPY (red curve) as a predictor of CFS, comparing CFS with healthy controls**. Larger values were associated with CFS cases. The 45 degree line (green) indicates the theoretical plot of a test with no discrimination between CFS and controls.

**Figure 3:**
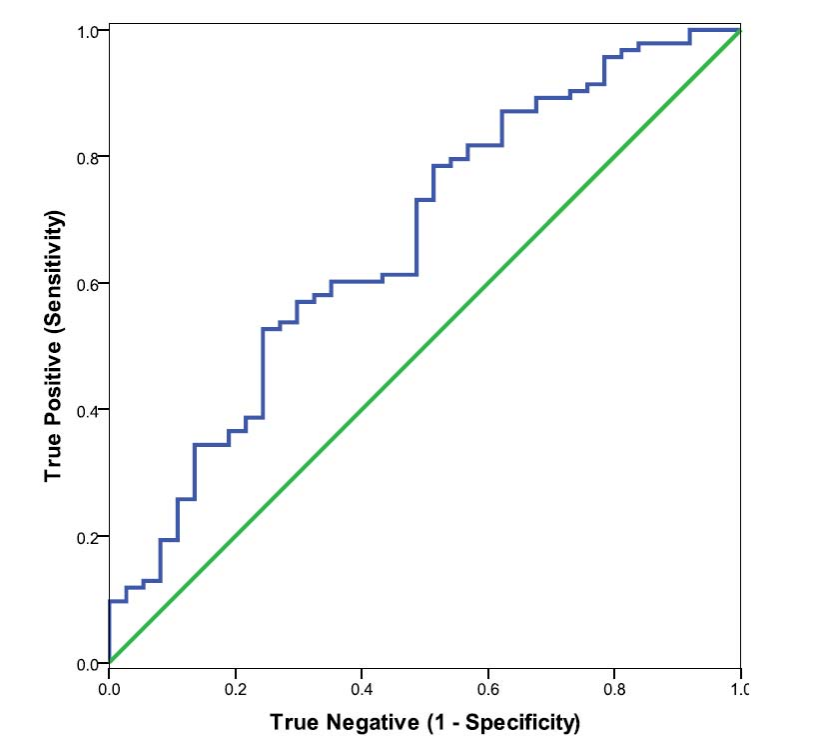
**ROC curve analyses of plasma NPY (blue curve) as a predictor of CFS, comparing CFS with GWI**. Larger values were associated with CFS cases. The 45 degree line (green) indicates the theoretical plot of a test with no discrimination between CFS and GWI.

Plasma NPY (pmol/L) was elevated in CFS subjects, compared to controls (mean for CFS patients = 102.4 +/-49.2, mean for controls = 79.8 +/-34.9, p = .000). CFS cases were also elevated when compared to GWI cases (mean for GWI patients = 75.1 +/- 38.1, p = .000). The distribution of plasma NPY values for the CFS cases, the healthy controls and for GWI are shown in Figure 1. ROC curve analyses of CFS and controls indicated that the predictive ability of plasma NPY was significantly better than chance alone (see Figure 2, Table 1). ROC curve analysis showed that NPY also distinguished GWI from CFS (Figure 3, Table 2). The coordinates of the curves (COC)) provide the entire spectrum of sensitivity/specificity pairs and a complete picture of test accuracy (see Additional files 1 and 2).

**Table 1:** Area Under the Curve (AUC): NPY in Plasma Comparing CFS and HC

AUC	Std.	**Asymptotic Sig.**^**b**^	Asymptotic 95%	Confidence Interval
	Error^a^		Lower Boundary	Upper Boundary
.655	.040	p < .000	.577	.733

^a^Under the nonparametric assumption

^b^Null hypothesis: true AUC = 0.5

**Table 2:** Area Under the Curve (AUC): NPY in Plasma Comparing CFS and GWI

Area	Std.	**Asymptotic Sig.**^**b**^	Asymptotic 95%	Confidence Interval
	Error^a^		Lower Bound	Upper Bound
.666	.053	p < .003	.561	.771

a. Under the nonparametric assumption

b. Null hypothesis: true area = 0.5

For 42 patients in this study we had both NPY and the results of a battery of clinical self report measures (Table 3). The plasma NPY had significant Pearson correlations with a variety of self report measures of symptom severity, including stress, negative mood and general health, and mental status in patients with CFS. The peptide also had Pearson correlations of p < 0.1 with measures of quality of life, vigor, emotional well-being, social functioning and fatigue disruption rating. In each case the correlation (+ or -) was in the anticipated direction. The demographic data for these 42 cases are shown in Table 4. The plasma NPY in this subset of CFS patients was elevated in comparison to healthy controls (p = .04) and to GWI cases (p = .05)

**Table 3:** Pearson Correlations (with p < 0.1) of Measurements Reflective of Symptom Severity and Plasma Neuropeptide Y in CFS cases

Instrument	Number	Pearson	Sig. (2-tailed)
	of Cases	Correlation	*p *Value
BECK Depression Inventory	42	.285*	.068
FSI Disruption Rating	42	.286*	.066
POMS Anger/Hostility Subscale	41	.490***	.001
POMS Confusion Subscale	42	.341**	.027
POMS Depression/Dejection Subscale	42	.349**	.024
POMS Friendly Subscale	42	-.562***	.000
POMS Vigor Subscale	42	-.294*	.059
Cope: Denial	42	.314**	.043
Cope: Behavioral Disengagement	42	.435***	.004
Cope: Self Blame	42	.315**	.042
ATQ Negative Self-Talk Score	42	.307**	.048
ATQ Positive Self-Talk Score	42	-.315**	.042
Perceived Stress Scale Total Score	42	.414***	.006
Quality of Life Total Score	42	-.297*	.056
SF-36 General Health	42	-.377**	.014
SF-36 Emotional Well-Being	42	-.264*	.091
SF-36 Social Functioning	41	-.281*	.075
CCSE-R Total Score	42	-.330**	.033

*p > .10; **p < .05; ***p < .01

**Table 4:** Description of CFS Cases in Table 3

Female	Plasma	Age	Age	Time since onset	Time since onset of	Sudden onset of
(%)	NPY(mean pmol/L +/- SD)	(range)	(mean)	of symptoms (range)	symptoms (mean +/- SD)	symptoms (%)
90%	97.4 +/- 31.2	23 - 60 years	46 years	1 - 38 years	12 +/- 8 years	66%

## Discussion

In this study, we found that the stress hormone, NPY, was statistically elevated in plasma from CFS cases compared to healthy controls - and to a group of patients with another fatiguing, multi-symptom illness, GWI. This later finding was unexpected. However, it might be explained by gender distribution, which was very different between CFS and GWI. ROC analysis indicated that plasma NPY has promise as a biomarker for distinguishing CFS both from healthy controls and .from related fatiguing illnesses. Our finding that NPY positively correlated with perceived stress, anger, depression, negative thoughts and maladaptive coping in patients with CFS suggests that NPY will be useful in defining subsets of patients for clinical trials and as a measure of therapeutic effects.

Abnormalities of the stress response are potential triggers or mediators of CFS symptoms [4]. Acute stress induces a fight-and-flight response that prepares the organism for coping with environmental challenge [47]. Sympathoadrenal activation during the stress response results in increased release of epinephrine (E) and norepinephrine (NE) from the adrenal medulla, increased NE and NPY release from the sympathetic nerve endings and changes in blood flow to a variety of organs [48]. Activation of the hypothalamic-pituitary-adrenal (HPA) axis results in increased secretion of glucocorticoids from the adrenal cortex [49].

Glucocorticoids regulate immune cell reactions to the stressor. In the brain, stress responsive neurotransmitter systems, in interaction with glucocorticoids, modulate affect, cognition and anxiety, and suppress behaviors that are inadequate for the situation, such as eating and reproduction. Increased noradrenergic activity in the brainstem increases vigilance and alertness and promotes memory for threatening stimuli [50]. These acute stress responses represent regulatory mechanisms that are critical to survival and adaptation of the species. Dysfunction in the regulatory capacity or interplay of these systems, as a potential consequence of dispositional risk or physical, chemical or emotional challenges, might plausibly exacerbate symptoms of CFS.

Immune activation and inflammation are postulated to be principle components in the pathophysiology of CFS [19,20,51]. Inflammatory responses are controlled by the HPA axis network that involves corticotropin-releasing hormone (CRH), adrenocorticotropic hormone (ACTH) and cortisol. Normally cortisol induces a down-regulation of inflammation. However, this mechanism is disrupted in the typically hypocortisolic CFS patient [52]. Broderick and colleagues propose that this disruption results not only from the possible failure of individual neuro-immune components but also involves a spontaneous restructuring of the control network [21]. Van Houdenhove and co-workers have formulated the hypothesis that the HPA axis hypofunction in CFS reflects a fundamental and persistent dysregulation of the neurobiological stress system [53]. Dysautonomic conditions (e.g., neurally mediated hypotension or orthostatic intolerance) have been reported in CFS patients as well as the related syndrome, GWI [54,55].

A recent study from our group demonstrated reduced stroke volume and cardiac output in more severely afflicted CFS patients [56]. Although we did not measure cortisol in the present study, we and others reported that moderate hypocortisolism occurs in CFS, and that it is of clinical relevance [57,58]. For example, CFS cases with low cortisol have a poorer response of CFS cases to cognitive behavioral stress management [58]. Of interest is the finding of Kempna, et al. [59], that NPY inhibits the production of cortisol in human adrenal H295R cells via the Y1 receptor. Antonijevic and colleagues showed that administration of NPY reduced cortisol secretion during night hours in healthy subjects [60]. In contrast, a series of experiments by Morgan, et al. demonstrated that acute, uncontrollable psychological stress elevated plasma NPY as well as plasma cortisol in healthy subjects [61,62].

Recently, our group reported that soluble as well as cell surface associated dipeptidyl peptidase IV (DPPIV) is decreased in CFS cases relative to controls [20]. NPY's biologic effects require interaction with its receptors. Native NPY 1-36 in the periphery is a major mediator of stress, responsible for prolonged vasoconstriction via Y1 receptors [29]. By cleaving the N-terminal Tyr-Pro dipeptide from NPY, DPPIV generates the Y2/Y5 receptor agonist NPY 3-36, that loses its affinity for the Y1 receptor and is angiogenic and inhibitory of NE release [30,63]. The low DPPIV observed in CFS coupled with high NPY would favor the Y1 receptor agonist form of NPY.

Another consideration of interest regarding NPY and CFS is the possibility of chronic viral infection in some patients. Du, et al [64], recently described increased NPY expression in the central nervous system (CNS) of mice following infection with a neurovirulent polytropic retrovirus. This virus infects macrophages and microglial cells resulting in production of proinflammatory cytokines [65], including interleukin 1 alpha (IL-1α), IL-β, and Il-6 known to be elevated in CFS [19]. Viral triggers such as EBV and HHV6 have long been suspected of involvement in the onset and persistence of CFS [3,5,6]. Evidence of xenotropic murine leukemia virus - related virus (XMRV) in peripheral blood mononuclear cells (PBMCs) in the majority of CFS cases, but not controls, supported the viral infection hypothesis [11]. This report by Lombardi, et al was followed by 4 subsequent reports of failure to detect any murine leukemia virus (MLV)-related virus gene sequences in blood from CFS patients [12,14,66,67] and one report of MLV-like virus gag gene sequences, but not XMRV, in 86.5% CFS cases compared with only 6.8% of healthy volunteer blood donors [13].

### Limitations

An obvious limitation of this study is that the samples represent a single point in time. We are presently conducting longitudinal studies. As Broderick and colleagues have pointed out, biomarkers measured in human subjects tend to be highly variable. These indicators are parts of a complex and integrated system and their inter-dependency must be addressed. The effect of medications such as cholesterol lowering, pain or anti-depression on plasma NPY is not known. However, cholesterol-lowering drugs are not well tolerated in CFS patients. Patients on opioid pain medications were excluded from the study. The effect storage of plasma for up to 5 years at -80°C on RIA detectible NPY is not known.

## Conclusions

This study is the first in the CFS literature to report that plasma NPY is significantly elevated over healthy controls and also over a comparision group, patients with GWI. The elevation of NPY in CFS cases is associated with severity of stress, negative mood and clinical symptoms. The pattern, in some CFS cases, of high NPY noted here, along with previously observed low DPPIV and hypocortisolism [20,57,58] supports the hypothesis of HPA axis dysregulation in CFS. Duration of this illness typically exceeds 10 years. Persistence is likely to involve complex interaction of immune, autonomic and neuroendocrine regulation and remains poorly understood. Investigation of CFS biology has focused on the detailed characterization of individual neuroendocrine and immune components taken in isolation. Current CFS treatments are directed at reducing symptom severity but no cure exists for this condition. In a review of CFS, published in Lancet, Prins, et al. stated: "Techniques such as bioimaging and proteomic strategies, and perhaps a systems biology approach, should be applied to try to elucidate such complicated interactions"[68]. It is clear that further understanding of disease mechanisms and development of effective treatments will require more than a list of the abundance of gene products, proteins or cells. These various cellular and molecular components are highly inter-dependent. Our research group has undertaken a systems biology approach [69]. We are presently incorporating data from plasma NPY measurements, along with other plasma and cellular biomarkers into a network analysis.

The renewed interest in viral infections in CFS suggests further studies. It is possible that NPY is induced by such infections. As reliable assays become available it will be important to determine the relationship of plasma NPY to potential pathogens.

## Competing interests

The authors declare that they have no competing interests.

## Authors' contributions

MAF and NGK conceived of the study, participated in its design and coordination, performed the statistical analysis and drafted the manuscript; GI and MA participated in study design; MR developed the NPY assay; NGK and SL participated in patients' diagnosis and assessment; ZB and JH participated in subject recruitment and data management; XRZ carried out the immunoassays; MAF, NGK, MA, SL and BH participated in cohort recruitment; GB evaluated the design and data analysis. All authors critically read and approved the final manuscript.

## Supplementary Material

Additional file 1 **Coordinates of the Curve CFS and HC**. The coordinates of the curves (COC) provide the entire spectrum of sensitivity/specificity pairs and a complete picture of test accuracy. Click here for file

Additional file 2 **Coordinates of the Curve CFS and GWI**. The coordinates of the curves (COC) provide the entire spectrum of sensitivity/specificity pairs and a complete picture of test accuracy. Click here for file
