# Supplementary material for: Plasma neuropeptide Y: a biomarker for symptom severity in chronic fatigue syndrome
Source: Behav Brain Funct. 2010 Dec 29;6:76. doi: 10.1186/1744-9081-6-76 (PMC3024290; doi:10.1186/1744-9081-6-76)
Supplement: Additional file 1 — Coordinates of the Curve CFS and HC. The coordinates of the curves (COC) provide the entire spectrum of sensitivity/specificity pairs and a complete picture of test accuracy. [file 1744-9081-6-76-S1.PDF]

Additional File 1. Coordinates of the Curve CFS and HC

Test Result Variable(s):Plasma NPY

Positive if Greater Than or Equal To[a]	Sensitivity	1 - Specificity
16.3410	1.000	1.000
21.5090	1.000	.990
26.8755	.989	.990
28.9405	.989	.980
30.0640	.978	.980
31.5285	.978	.970
34.8130	.978	.960
37.3380	.968	.960
38.4580	.968	.950
39.6000	.968	.940
40.2550	.968	.930
40.8390	.968	.920
42.2805	.968	.910
44.9800	.957	.910
46.9320	.957	.900
47.2735	.957	.890
47.6925	.957	.880
48.2850	.946	.880
49.0085	.935	.880
49.6035	.925	.880
50.2720	.914	.880
51.0320	.914	.870
51.4435	.903	.870
52.1115	.892	.870
52.7160	.892	.860
52.9725	.892	.850
53.4210	.892	.840
53.7870	.892	.830
54.2540	.882	.830
54.7485	.882	.820
54.9680	.882	.810
55.3070	.882	.800
55.8750	.871	.800
56.4790	.871	.790
56.7350	.871	.780
56.8175	.871	.770

56.9680	.871	.760
57.2350	.860	.760
57.4415	.860	.750
57.5725	.860	.740
57.7010	.860	.730
57.8890	.849	.730
58.0140	.839	.730
58.1800	.839	.720
58.6590	.828	.720
59.1245	.828	.710
59.3350	.817	.710
60.2325	.817	.690
61.1025	.817	.680
61.2215	.817	.670
61.5870	.817	.660
61.8890	.806	.660
62.1890	.796	.660
62.6000	.796	.650
62.8410	.785	.650
63.1900	.774	.650
63.7210	.774	.640
64.0690	.774	.630
64.6220	.763	.630
65.2625	.763	.620
65.8625	.763	.610
66.4300	.753	.610
66.7150	.742	.610
67.0100	.742	.600
67.1650	.731	.600
67.5845	.731	.590
68.0240	.720	.590
68.6345	.720	.570
69.2475	.720	.560
69.3695	.720	.550
70.0805	.710	.550
71.0965	.710	.540
71.5300	.710	.530
71.7225	.699	.530
71.8875	.699	.520
72.1170	.688	.520

72.3915	.677	.520
72.8210	.667	.520
73.2145	.667	.510
73.3350	.656	.510
73.4910	.645	.510
73.9380	.645	.500
74.3520	.645	.490
74.6550	.634	.490
75.1650	.634	.480
75.5395	.624	.480
75.7845	.624	.470
75.9585	.613	.470
76.3825	.613	.460
77.5085	.613	.450
78.6255	.613	.440
79.6875	.613	.430
80.4565	.613	.410
81.2625	.613	.400
82.8645	.602	.400
84.0545	.602	.390
84.6220	.602	.380
85.6380	.602	.370
86.7185	.602	.360
87.0390	.591	.360
87.2585	.581	.360
87.4875	.581	.350
87.6250	.581	.340
88.0005	.570	.340
88.5500	.570	.330
88.8980	.570	.320
89.1450	.570	.310
90.0235	.570	.300
90.8200	.559	.300
90.9485	.548	.300
91.1775	.548	.290
91.6390	.548	.280
91.9475	.538	.280
92.0075	.538	.270
92.1660	.538	.260
92.3560	.538	.250

92.7495	.538	.240
93.3095	.527	.240
93.6485	.516	.240
94.0800	.505	.240
94.5570	.495	.240
95.0495	.495	.230
95.3965	.484	.230
95.5630	.473	.230
96.1030	.462	.230
96.5240	.462	.220
96.7805	.452	.220
97.2935	.452	.210
97.9945	.441	.210
98.5710	.430	.210
98.8310	.430	.200
99.3895	.430	.190
100.6345	.419	.190
101.9600	.419	.180
102.6745	.409	.180
103.1595	.398	.180
103.4800	.387	.180
103.7570	.387	.170
104.0870	.376	.170
104.1970	.366	.170
104.6085	.366	.160
105.5015	.366	.150
106.0325	.355	.150
106.4575	.355	.140
107.1450	.355	.130
108.2070	.344	.130
109.6720	.344	.120
110.6350	.333	.120
111.8350	.323	.120
113.1425	.312	.120
113.7450	.301	.120
116.1990	.301	.110
119.5515	.301	.100
121.0150	.290	.100
121.8850	.280	.100
122.7850	.269	.100

125.7150	.258	.100
128.2950	.258	.090
128.6250	.258	.080
129.2110	.247	.080
129.9130	.247	.070
130.8470	.237	.070
131.3950	.237	.060
131.5250	.226	.060
132.0650	.215	.060
132.8700	.204	.060
133.3000	.194	.060
134.2050	.194	.050
136.8750	.194	.040
138.7350	.183	.040
139.1980	.172	.040
141.1740	.172	.030
143.2250	.161	.030
143.9140	.151	.030
144.4265	.140	.030
146.1115	.129	.030
150.3950	.118	.030
155.4650	.108	.030
160.9300	.097	.030
165.5900	.097	.020
168.4000	.086	.020
178.5070	.075	.020
189.0670	.075	.010
200.2900	.065	.010
217.5095	.054	.010
229.0100	.043	.010
234.4850	.032	.010
245.9655	.022	.010
255.6700	.011	.010
262.3890	.000	.010
269.9800	.000	.000

a. The smallest cutoff value is the minimum observed test value minus 1, and the largest cutoff value is the maximum observed test value plus 1. All the other cutoff values are the averages of two consecutive ordered observed test values.
