# Supplementary material for: Plasma neuropeptide Y: a biomarker for symptom severity in chronic fatigue syndrome
Source: Behav Brain Funct. 2010 Dec 29;6:76. doi: 10.1186/1744-9081-6-76 (PMC3024290; doi:10.1186/1744-9081-6-76)
Supplement: Additional file 2 — Coordinates of the Curve CFS and GWI. The coordinates of the curves (COC) provide the entire spectrum of sensitivity/specificity pairs and a complete picture of test accuracy. [file 1744-9081-6-76-S2.PDF]

Additional File 2. Coordinates of the Curve: CFS and GWI

Variable(s):NPYplasma,T1

Positive if Greater Than or Equal To[a]	Sensitivity	1 - Specificity
.6000	1.000	1.000
6.8200	1.000	.973
15.3500	1.000	.946
22.1685	1.000	.919
27.7420	.989	.919
29.9435	.978	.919
32.4700	.978	.892
34.8950	.978	.865
35.9100	.978	.838
40.0900	.968	.838
43.3065	.968	.811
44.6215	.957	.811
46.9925	.957	.784
48.2850	.946	.784
49.0085	.935	.784
49.6035	.925	.784
50.1425	.914	.784
50.9025	.914	.757
51.2175	.903	.757
51.4460	.903	.730
52.3760	.892	.730
53.4000	.892	.703
53.7580	.892	.676
54.6295	.882	.676
55.5715	.871	.676
55.7100	.871	.649
56.4200	.871	.622
57.4350	.860	.622
57.8890	.849	.622
58.1740	.839	.622
58.8055	.828	.622
59.9055	.817	.622
60.5550	.817	.595

61.2200	.817	.568
61.8890	.806	.568
62.0740	.796	.568
62.4850	.796	.541
62.8250	.785	.541
62.9360	.785	.514
63.5380	.774	.514
65.2220	.763	.514
66.4300	.753	.514
66.8350	.742	.514
67.4400	.731	.514
67.8595	.731	.486
68.7015	.720	.486
70.5140	.710	.486
71.7590	.699	.486
72.1170	.688	.486
72.3915	.677	.486
72.8665	.667	.486
73.3350	.656	.486
73.9050	.645	.486
74.9100	.634	.486
75.6650	.624	.486
78.5650	.613	.486
81.2500	.613	.459
81.6375	.613	.432
83.5625	.602	.432
85.6800	.602	.405
86.2900	.602	.378
86.6900	.602	.351
87.0390	.591	.351
87.1690	.581	.351
87.5170	.581	.324
88.4970	.570	.324
89.9800	.570	.297
90.8200	.559	.297
91.4100	.548	.297
91.9450	.538	.297
92.5545	.538	.270
93.2545	.527	.270
93.4550	.527	.243

93.6485	.516	.243
94.0800	.505	.243
94.8655	.495	.243
95.3965	.484	.243
95.5630	.473	.243
96.1120	.462	.243
97.0460	.452	.243
97.9945	.441	.243
99.1295	.430	.243
101.1545	.419	.243
102.6745	.409	.243
103.1595	.398	.243
103.6900	.387	.243
103.9670	.387	.216
104.0870	.376	.216
104.2150	.366	.216
105.1350	.366	.189
106.7200	.355	.189
107.7050	.344	.189
108.7650	.344	.162
109.9550	.344	.135
110.6350	.333	.135
111.8350	.323	.135
113.1425	.312	.135
117.0975	.301	.135
121.0150	.290	.135
121.8850	.280	.135
122.7850	.269	.135
123.6900	.258	.135
126.5750	.258	.108
129.6320	.247	.108
130.8820	.237	.108
131.5250	.226	.108
132.0650	.215	.108
132.8700	.204	.108
135.2600	.194	.108
138.0000	.194	.081
138.7350	.183	.081
140.7360	.172	.081
143.2250	.161	.081

143.9140	.151	.081
144.4265	.140	.081
145.3615	.129	.081
146.7100	.129	.054
148.3100	.118	.054
151.2450	.118	.027
155.4650	.108	.027
157.6750	.097	.027
162.3350	.097	.000
168.4000	.086	.000
180.4400	.075	.000
200.2900	.065	.000
217.5095	.054	.000
229.0100	.043	.000
234.4850	.032	.000
245.9655	.022	.000
255.6700	.011	.000
256.7980	.000	.000

a. The smallest cutoff value is the minimum observed test value minus 1, and the largest cutoff value is the maximum observed test value plus 1. All the other cutoff values are the averages of two consecutive ordered observed test values.
